# Frequency of salivary flow rate measurements in Sweden 2008–2024

**DOI:** 10.2340/aos.v85.46503

**Published:** 2026-07-16

**Authors:** Håkan Flink, Anders Hedenbjörk-Lager, Eva Nohlert, Åke Tegelberg

**Affiliations:** a Centre for Clinical Research Västmanland, Uppsala University, Västerås, Sweden; b Cariology, Faculty of Odontology, Malmö University, Malmö, Sweden

**Keywords:** Xerostomia, hyposalivation, saliva flow rate measurement, caries prevention, medication

## Abstract

**Objective:**

Saliva flow rate measurement (SFRM) is a prerequisite for accurate diagnosis of hyposalivation, but national data about the frequency of SFRM conducted in the Swedish population are lacking. The objective was to describe performed SFRMs in the population via registration in the Swedish dental financial system and compare with the earlier reported prevalence of hyposalivation.

**Material and Methods:**

A descriptive retrospective registry-based study was completed. Dental care registry data from 2008 to 2024 were investigated to determine the proportion of individuals > 25 years of age who had undergone SFRM.

**Results:**

During 2008–2024, 70,390 SFRMs were conducted in Sweden. SFRMs began to increase in 2013. SFRMs were performed more than twice as often for females than males. Measurements increased by age up to a peak at 65–69 years of age (1.7%) and decreased thereafter. The frequency of SFRM amounted to approximately 0.9% of the total Swedish population.

**Conclusions:**

The number of SFRMs performed in the Swedish population is low overall, with increased numbers in women and in older ages. The findings support that an increased use of SFRM to improve diagnosis of hyposalivation could be beneficial to understand more about the prevalence of hyposalivation and support for preventive caries treatment.

## Introduction

An increasing proportion of older people need dental care. During the life course, many will also need some kind of medication, frequently causing xerostomia – the subjective feeling of dry mouth – as a side effect. Xerostomia will, in turn, increase the caries risk, especially as a greater proportion of older people have more remaining teeth than previous generations [1].

Recent data from the Swedish Quality Registry for Caries and Periodontal Disease (SKaPa) on caries prevalence in a life course perspective show an increase in caries experience in older age groups [2].

A reformed Swedish national dental insurance system for adults was introduced in 2008. The aim was to enhance oral health through regular dental care and prevention, thus contributing to more affordable costs for patients. A general dental care subsidy, Allmänt Tandvårdsbidrag (ATB), was introduced in tandem with a system for high-cost protection [3]. A special dental care subsidy, Särskilt Tandvårdsbidrag (STB), for patients with special conditions or disabilities that could result in increased risk for impaired oral health, was added in 2013 [4].

STB can only be used for dental check-ups or preventive measures such as professional dental cleaning. For some medical conditions, the prerequisites for STB are hyposalivation caused by long-time medication, radiation therapy to the head or neck, or Sjögrens syndrome. To receive the subsidy, a medical certificate is needed, in combination with a saliva flow rate measurement (SFRM). SFRM is a quantitative chairside tool for the diagnosis of hyposalivation and has been used for caries risk assessment (CRA) for many years prior to the introduction of STB [5, 6]. An accurate CRA will provide the clinician with essential knowledge to enable them to design a treatment plan that includes sufficient preventive and nonoperative measures [7, 8].

The hypothesis of this study was that the ratio between performed SFRMs and the earlier described prevalence of hyposalivation in the population is not in proportion.

Thus, the study aim was to describe the frequency of performed SFRMs in the population as registered in the Swedish dental financial system, approximately 4 million individuals yearly, 4,265,238 in 2025 [9].

## Material and methods

### Study design

This is a descriptive retrospective registry-based study, based on dental care data from the years 2008–2024 as registered at the Swedish Social Insurance Agency (Försäkringskassan, FK) [9]. Data and figures are used with permission from FK.

### Study population

The study population consisted of individuals in Sweden older than 25 years of age who had undergone SFRM registered by the national FK between 2008 and 2024.

### SFRM

A description of the SFRM procedure and corresponding treatment code (161) is published by the Dental and Pharmaceutical Benefits Agency (Tandvårds-och läkemedelsförmånsverket, TLV) [10]. According to the description, the patient is given a moment to rest before the measurement. The SFRM includes both unstimulated and stimulated whole saliva [4]. To qualify for an STB subsidy, both salivary flow rates need to be low, that is, an unstimulated saliva flow rate of 0.1 mL/min or less for a 15-min measurement and a stimulated saliva flow rate of 0.7 mL/min or less for a 5-min measurement.

### Variables and statistics

Data were collected on the number of SFRMs performed yearly (code 161), age group and sex of patients, and the category of caregiver (public or private care) when performing the SFRM.

Full-grade assessment was not feasible due to heterogeneity and limited number of studies.

The Swedish Ethical Review Authority approved the research project (Dnr 2025-06898-01).

## Results

**Figure 1:**
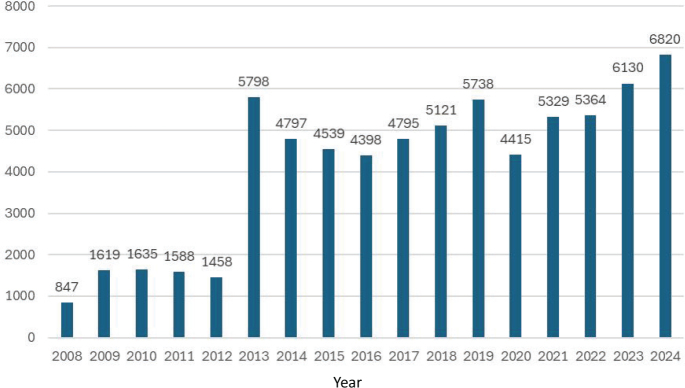
Number of saliva flow rate measurements (SFRMs) performed per year from 2008* to 2024 in Sweden. * In 2008, the SFRMs started on July 1. Total SFRMs: 70,390.

Over the period 2008–2024, 70,390 SFRMs were conducted in Sweden. The frequencies per year are illustrated in Figure 1. The total Swedish population older than 25 years of age is circa 7.5 million [11], giving an SFRM rate of 0.9%. A mean of 4,140 SFRMs were performed each year during the observation period. The renewed national dental insurance system for adults was introduced on July 1, 2008. Thus, the data cover only 6 months of the first year [12]. The STB was introduced on January 1, 2013, resulting in an increase in performed SFRMs. In 2020, the number of SFRMs decreased, corresponding to reduced numbers of dental visits during the COVID-19 pandemic. Since then, there has been a continued increase.

**Figure 2:**
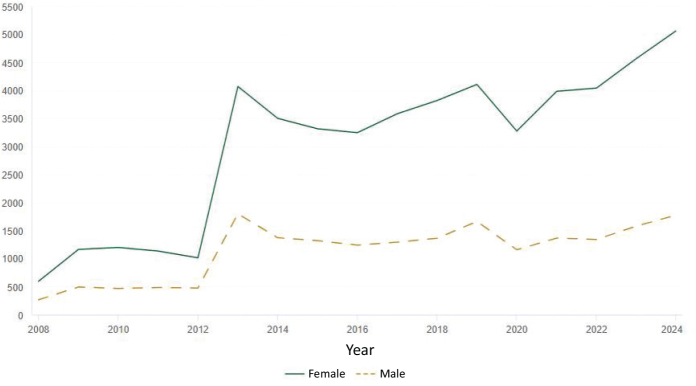
Number of saliva flow rate measurements (SFRMs) performed per year from 2008* to 2024, divided by sex in Sweden. * In 2008, the SFRMs started on July 1. Total SFRMs: 70,390.

**Figure 3:**
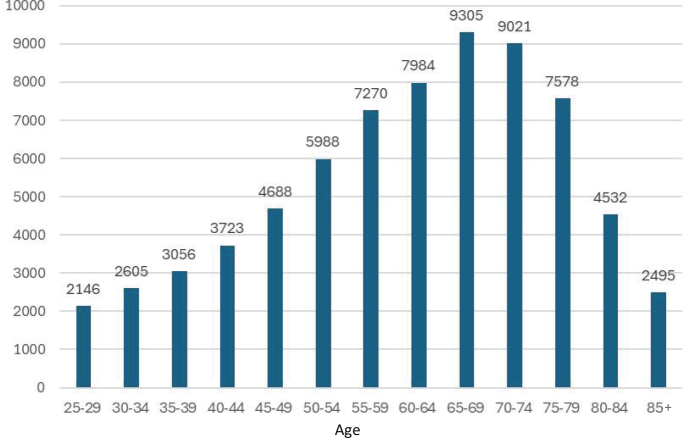
Number of saliva flow rate measurements (SFRMs) performed per year from 2008* to 2024 in Sweden, divided by the age when measurement was performed. * In 2008, the SFRMs started on July 1. Total SFRMs: 70,390. The figure shows absolute counts as calculation of population-based rates was impossible.

There was a distinct sex difference; SFRMs were performed more than twice as often for females than males (Figure 2). Analysis of SFRMs stratified by patient age showed an increase with age, with a peak at 65–69 years of age. Thereafter, a decrease in the number of SFRMs was observed (Figure 3). In the age group with the most frequent test rate (65–69-year-olds), 9,305 SFRMs were performed in a population of 548,406 individuals (2024), equating to approximately 1.7% [11].

**Figure 4:**
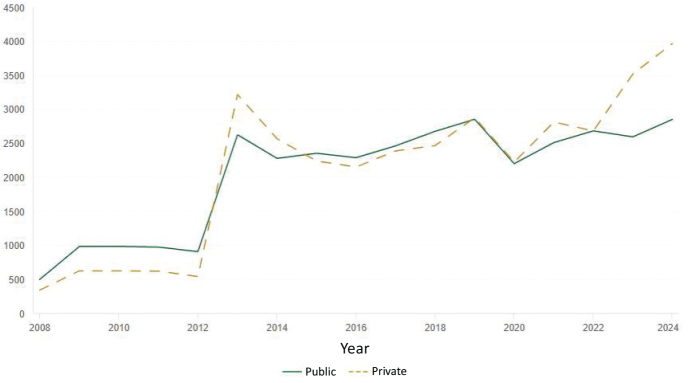
Number of saliva flow rate measurements (SFRMs) performed per year from 2008* to 2024 in Sweden, by category of caregiver (public care or private care companies). * In 2008, the SFRMs started on July 1. Total SFRMs: 70,390.

There were no differences in SFRM frequency between public and private dental caregivers until 2023, when a slight increase was observed among private dental caregivers (Figure 4).

## Discussion

This descriptive retrospective registry-based study is the first study describing the national frequency of annually performed SFRM over a longer period.

The main finding was that the frequency of performed SFRMs was low in the Swedish population aged 25 years and older who had visited dental care, but increased from 2013 onward, which seemingly coincides with the introduction of STB.

There could be several possible reasons for this increase. Firstly, STB was announced as a potential financial benefit for the patient, amounting to an annual reduction of approximately 110 euros in dental fees. Secondly, the inclusion of STB created possibilities for dental practitioners to aid patients by diagnosing hyposalivation and may also have contributed to an increased awareness of the problem. Thirdly, patients may have received external information about STB and SFRM and awareness of the possibility of getting a subsidy might have encouraged them to seek help for their dry mouth problems, which in turn could explain their perception of xerostomia and caries activity, possibly leading to an increased patient demand for SFRMs.

A key strength of this study is that it includes all SFRMs performed within the social insurance system (FK) in Sweden over a 17-year period.

The study is descriptive and exploratory, and causal inferences are not possible. It just describes the frequency of a procedure, not diagnostic outcomes, clinician behaviors or motivation, patient attitudes, subsidy uptake behaviors, or other systemic factors (e.g. guideline changes, awareness, registry modifications).

We do not know if some individuals may have made multiple SFRM, but due to the cost of testing (85 Euro, 2025) and doubtful gains of information by multiple SFRM, this may be less likely. However, if repeat measurements are included, the proportion of unique individuals tested may be even lower than reported.

Another limitation is that the actual prevalence of diagnosed hyposalivation among those tested is still unknown. The latest larger estimated prevalence of hyposalivation in 1995 found to be was around 20–30% for the 65–69-year-olds age group [13].

This shortcoming indicates a future need for a specific diagnosis code for hyposalivation as well as for xerostomia in the dental care system. Such codes would allow register-based prevalence analyses and constitute an important possibility for future research as the prevalenc of hyposalivation and xerostomia in older people varies widely (10–75%) between existing studies [14–16]. As this is a first descriptive and exploratory study of national prevalence of performed SFRM, further studies are required about regional variations, socioeconomic stratification as well as analysis of urban versus rural differences.

Further limitations are the registry-based nature of the data, where there is a lack of diagnostic outcome information, absence of clinical variables (e.g. medication use, xerostomia severity), potential miscoding, and lack of data on privately performed SFRMs without financial reimbursement.

The discrepancy between the estimated prevalence of hyposalivation in the population and the frequency of performed SFRMs needs attention. Reduced saliva secretion is a very important negative factor for several oral health conditions, such as dental caries, erosive tooth wear, oral clearance of food debris, oral mucosal health, periodontal health, the sense of taste, the ability to masticate and swallow food, and general oral comfort, including social interaction.

A sex difference covering the entire observation period was discovered (Figure 2). This was likely a reflection of the previously described sex difference regarding the prevalence of both hyposalivation and xerostomia [14]. This observation based on absolute counts needs to be adjusted to population data. It is unclear if this sex difference corresponds to the reported higher incidence of decayed, missing and filled teeth (DMFT) in 60 years and over in 2012 and 70 years and older women in 2022 [17], which needs further research.

When the number of SFRMs performed by age group was considered (Figure 3), an increase with age, which peaked at 65–69 years, was observed. The age-related SFRMs probably also reflect the varying prevalence of hyposalivation and xerostomia in different age groups [18]. The pattern of SFRMs in different age groups may also mirror the recently described prevalence of caries experience [2], but this theoretical relationship needs further investigation. The decline in SFRMs in the 70–74-year and older age groups may indicate an age-related decline in general health [19], which could be exemplified by a declining pattern of dental examinations with age [20]. These absolute counts in different age groups need to be population adjusted for age-specific rates to ensure if it reflects increased diagnostic activity or simply demographic structure.

The slight increase in SFRMs among private dental caregivers in the last 2 years of registration time may reflect the higher proportion of older patients among their patients compared with patients of public dental caregivers [9]. During the observation period, private dental caregivers performed approximately two-thirds of all dental check-ups among adults in the Swedish population [9].

It has been presumed that SFRMs are seldom conducted in general dental practice, but descriptive data have been lacking. In the age group with the most frequent test rate (65–69-year-olds), approximately 1.7% [11] had undergone SFRM, which is in contrast with the estimated prevalence of hyposalivation was around 20–30% for the same age group in 1995 [13]. Consequently, the diagnosis of hyposalivation in the population may likely be underestimated. There might be several reasons for underuse of this diagnostic tool. The cost for the SFRM procedure might be considered too high or perceived not to provide enough benefits by the patients. This is especially true for patients with a negative opinion about paying for prevention [21], perhaps as a consequence of reoccurring caries when previous attempts at preventions have failed [2]. Awareness about the prevalence of hyposalivation and xerostomia is low among dental professionals [22] and a knowledge gap exists regarding diagnosis of hyposalivation and the subsequent treatment strategies aimed at managing the problem [23]. Dentists are not always interested in caries prevention and may also be, at least to some extent, unaware of the available strategies for patients with hyposalivation and reoccurring caries [21]. This lack of knowledge on caries prevention within the dental profession undoubtedly creates unnecessary obstacles for affected patients seeking information about what may help them. Where hyposalivation and xerostomia are not diagnosed by the dental profession, alternative identification of xerostomia in relation to medications has been reported in primary care settings, albeit with a very low detection rate 0.23% [24].

The results of this study describe a ‘Know–Do Gap’ problem, where it is known that many medications can cause hyposalivation, but despite this knowledge, the dental professionals do not implement either the required diagnostics or the caries preventive efforts [25]. Recent Swedish guidelines for evidence-based interventions regarding dental caries [26] as well as xerostomia [27] recommend SFRM on a perceived basis of individual need. These recommendations might be too ambiguous if an increase in SFRMs is desired. More precise instructions regarding the indications and clinical procedures when performing an SFRM could be of benefit [10].

Clinical consequences and implementation gap may relate to unclear Swedish clinical guidelines that do not explicitly define when SFRM should be performed, how the results should be integrated into CRA, or which preventive or therapeutic measures should follow a confirmed diagnosis. When the performed SFRM does not lead to clearly defined and economically supported management pathways, this may represent a relevant explanatory factor for its limited uptake.

The diagnosis of hyposalivation is important for accurate CRA of the patients and provides crucial information for clinicians to recommend and provide sufficient prevention strategies [7, 8]. To encourage and develop sufficient caries prevention for patients with hyposalivation, monitoring the implementation of SFRMs and adding diagnosis codes for hyposalivation and xerostomia may help. This could facilitate follow-up of caries preventive efforts (by specific codes) recommended in real-world data [28] and could further indicate potential benefits for patients, clinicians, and stakeholders. These clinical implications should, however, be interpreted with caution and in the context of limited data.

## Conclusion

The low frequency of SFRMs performed in Sweden indicates underuse of a valuable diagnostic tool and a need for an increase in SFRMs to be able to accurately diagnose cases of hyposalivation to a higher degree, especially in female and older patients.
